# Wheat Bran‐Derived Carbohydrates as Functional Food Ingredients: Extraction and Evaluation of Prebiotic Potential

**DOI:** 10.1111/1750-3841.71280

**Published:** 2026-07-09

**Authors:** Débora Regina Magro, Maria Eduarda Cordeiro Silva, Deisy Alessandra Drunkler, Flavio Dias Ferreira, Eliane Colla

**Affiliations:** ^1^ Graduate Program in Food Technology (PPGTA), Department of Food Federal Technological University of Paraná Medianeira Paraná Brazil; ^2^ Department of Food Federal Technological University of Paraná Medianeira Paraná Brazil

**Keywords:** arabinoxylans, prebiotic potential, probiotics

## Abstract

Wheat bran (WB) is a rich source of non‐starch polysaccharides, particularly arabinoxylans, which can be valorized into functional food ingredients with potential prebiotic properties. This study investigated the extraction of carbohydrates from WB using ultrasonic and hydrothermal treatments and evaluated their technological and functional characteristics and in vitro prebiotic potential. The extraction yields reached 43.47% and 72.36% for ultrasonic and hydrothermal methods, respectively. The extracted carbohydrates exhibited low in vitro digestibility (0.86%–2.04%), indicating resistance to gastrointestinal conditions. Fermentation assays demonstrated selective stimulation of probiotic strains, including Lactobacillus acidophilus, Lactobacillus paracasei, Lactobacillus rhamnosus, and Bifidobacterium lactis, accompanied by increased sugar consumption and organic acid production. Prebiotic activity score (PAS) evaluation showed the strongest responses for Lactobacillus acidophilus, particularly in relation to *Salmonella* Typhimurium, whereas more limited responses were observed for the remaining probiotic strains and enteric comparisons. These results suggest that WB‐derived carbohydrates exhibit prebiotic potential properties and may represent candidate prebiotic ingredients, although further in vivo studies are required to confirm prebiotic effects.

## 1 Introduction

The growth in food production is directly accompanied by an increase in agro‐industrial waste resulting from processing. These residues are inherent to the processes, and many arise from the need to exclude components that do not integrate into the products, such as bran, husks, and stones, and, through technological strategies, become by‐products. Many of these by‐products are used to make animal feed; however, researchers have been looking for other applications because they are low‐cost, have a high nutritional value, and, when incorporated into formulations, can add commercial and nutritional value to the final product (Alencar et al. 2020).

According to the Food and Agriculture Organization (FAO 2025), wheat is the most consumed food in the world; its global production in 2024 was 792.2 million tons. The product from milling wheat grain is 25% bran and 75% flour. According to ABITRIGO (Brazilian Wheat Industry Association), in 2024, Brazil milled 13.19 million tons of wheat, generating 3.29 million tons of bran and 9.89 million tons of flour (ABITRIGO 2024).

Wheat bran (WB) has been used for animal feed and in the brewing industry due to its high carbohydrate content and nutritional value. Its composition varies according to the variety of wheat, seasonality, and technological process, with approximately 9.6% to 18.6% protein, 3% to 4% fat, 3% to 8% minerals, and 60% to 75% carbohydrates; of these, 9.1% to 38.9% are starch and 55% to 60% are non‐starch carbohydrates, based on dry matter. Among the non‐starch carbohydrates, the main one is arabinoxylan (AX; 36.5%). Still, they also contain cellulose (11%), lignin (3% to 10%), and uronic acids (3% to 6%) (Saini et al. 2023; Shang et al. 2021).

Most AXs are incorporated into the cell wall matrix and intertwined with other macromolecules, which makes them difficult to extract with water. Many authors have used different methodologies to extract them. Kong et al. (2023) used enzymes and extrusion methods to achieve the highest AX content of AXs in WB.

Broekaert et al. (2011) and Van et al. (2008) observed that AX can be hydrolyzed by WB‐associated endoxylanases or by specific intestinal bacteria that produce these enzymes, leading to the formation of arabinoxylooligosaccharides (AXOS). These oligosaccharides have prebiotic properties and promote health by stimulating the activity of beneficial bacteria in the intestinal colon.

Paesani et al. (2024) highlight the potential of WB as a food supplement due to its low production cost and high fiber content, for which there is already evidence of reduced risk of certain chronic diseases and a prebiotic effect. These characteristics are increasingly sought after by consumers, who are increasingly concerned about health and healthy eating habits. A growing trend is using symbiotic solutions of prebiotics and probiotics incorporated into new products, such as yogurts, infant formulas, skimmed creams, chocolates, and bakery products (Dzah et al. 2020).

This new consumer profile has increased demand for functional foods, new ingredients, innovations from industry and research centers, and sustainability. Therefore, the search for prebiotic foods, in line with more sustainable extraction methodologies with low environmental impact and cost, has been gaining ground, including hydrothermal and ultrasound extraction. Both methodologies stand out for their reduced consumption of organic solvents, as they do not use catalysts or chemicals that generate corrosion problems (Antunes et al. 2023).

Hydrothermal treatments, including steam explosion and mainly liquid hot water (autohydrolysis), are a potential low‐impact treatment technology that is used to extract hemicellulosic materials in which polysaccharides undergo hydrolysis at high temperature and pressure, usually using an autoclave (Ruiz et al. 2023).

Ultrasound can be applied using a probe or by immersion in ultrasound baths. Cavitation is the main effect generated by ultrasound; it generates energy and causes disturbances in plant cells, alters their chemical and physical properties, produces shear forces capable of breaking polymer chains, and facilitates the release of cell‐matrix components (Ruiz et al. 2023; Wang et al. 2024).

Research indicates that ultrasound has been widely used to improve yields in processes for extracting carbohydrates from biomass, with studies examining process variables such as ultrasound energy, operating amplitude, temperature, treatment duration, and sample concentration (Thirunavookarasu et al. 2024).

Considering this context and the demand for more straightforward, cost‐efficient extraction techniques, this study aimed to investigate the extraction of carbohydrates from WB using ultrasonic and hydrothermal treatments and to characterize their in vitro prebiotic potential.

## 2 Materials and Methods

### 2.1 Raw Material, Microorganisms, and Reagents

The WB was donated by a mill in the western region of Paraná and stored at −18°C in a domestic freezer. The probiotic microorganisms *Lactobacillus* *acidophilus (*Howaru Dophilus LYO), *Lacticaseibacillus paracasei* (L. casei‐01)*, Lacticaseibacillus rhamnosus* (Nu‐trish, LGG), *Bifidobacterium lactis* HN019 (Howaru Bifido LYO), and the prebiotics oligofructose (Orafti P95) and inulin (Orafti GR) were obtained from companies specialized in the field; the pathogens (*Escherichia coli* ATCC 43888 and *Salmonella* Typhimurium ATCC 14028) were from the American Type Culture Collection (ATCC). The enzymes used to simulate gastrointestinal digestion, as well as the sugar and acid standards, were purchased from Sigma‐Aldrich. All the reagents used were of analytical grade, and the culture media were of microbiological standard.

### 2.2 WB's Centesimal Composition

The WB was characterized according to the official methods of AOAC (2023) for moisture (Method 925.45b), ash (Method 923.03), proteins (Method 960.52; N x 5.95), lipids (Method 920.39), and carbohydrates by difference.

### 2.3 Study of Methods for Extracting Carbohydrates From WB by Ultrasonic and Hydrothermal Treatment

In evaluating extraction methods, a sequential experimental design was used. For ultrasound‐assisted extraction, a Central Composite Rotatable Design (CCRD 2^3^) was initially employed to investigate the effects of solids load (30–100 g L^−^^1^), ultrasonic probe power amplitude (20%–80%), and extraction time (3–10 min). Subsequently, a second CCRD (2^2^) was performed, considering solids load (20–50 g L^−^^1^) and extraction time (10–15 min), with the ultrasonic probe amplitude fixed at 80%. The experiments were carried out by dispersing WB in distilled water in a jacketed 1 L beaker, with the sonication probe (diameter of 25.4 mm) immersed in the suspension. The extraction temperature was controlled at 15°C by circulating water from a thermostatic bath through the jacketed vessel. Ultrasound treatment was performed using an ultrasonic probe (Vibra Cell VC 50, SONICS, USA) operating at 20 kHz, with a nominal power of 350 W.

For the hydrothermal extraction, a CCRD 2^3^ was also used to evaluate the effects of solids load (30–100 g L^−^^1^), pH (3–7), and extraction time (10–60 min). Subsequently, a CCRD 2^2^ was conducted using solids load (20–50 g L^−^^1^) and pH (3–5), with the extraction time fixed at 30 min. The experiments were carried out by suspending WB in distilled water in 1.0 L Erlenmeyer flasks, followed by pH adjustment with 1.0 M HCl (solids load and pH for each run), in an autoclave (AV‐18, Phoenix, Araraquara, SP, Brazil) at 121°C (0.11 MPa).

After the extraction processes, the supernatants were recovered by centrifugation (Routine 420 R, Hettich, Kirchlengern, Germany) at 5000 × g for 5 min at 25°C. The total carbohydrate content in the extracts (g CHO.g^−^^1^ WB) was determined by the Anthrone method (glucose 0.1 g L^−^^1^ as standard, absorbance measured at 620 nm). The extraction yield was subsequently calculated according to Equation (1) (Osborne and Voogt 1985).

(1) $$\mathrm{Extract}\;\mathrm{yield}\;\left(\%\right)=\frac{\%\;\mathrm{CHO}\;\mathrm{of}\;\mathrm{extract}}{\%\;\mathrm{CHO}\;\mathrm{of}\;\mathrm{WB}}.\;100$$

### 2.4 Precipitation and Characterization of Carbohydrates Extracted From WB

The carbohydrates extracted by the most favorable extraction conditions, present in the collected supernatants (2.3), were precipitated in 80% ethanol (1:1 v.v^−1^) and stored at 4°C overnight, followed by centrifugation (Routine 420 R, Hettich, Kirchlengern, Germany). No washing steps were applied after precipitation to remove low‐molecular‐weight sugars (the recovered fraction should be considered a mixture of WB‐derived carbohydrates). Subsequently, the precipitate was freeze‐dried (Labconco, FreeZone, USA) and ground to an average particle size of 70 mesh to obtain the CWB.

The functional groups of extracts were analyzed by Fourier transform infrared spectroscopy (FTIR) using a spectrometer (WB‐IR Spectrum 100S, PerkinElmer, Waltham, MA, USA) equipped with an attenuated total reflectance accessory. FTIR spectra of the samples were recorded over 600–4000 cm^−1^ with a resolution of 4 cm^−1^. FTIR spectra were obtained using the OriginPro 10.2 program (OriginLab Corporation, Northampton, MA, USA).

The freeze‐dried and ground CWB was hydrolyzed according to the ASTM E1758‐1 methodology (ASTM 2020) and analyzed by high‐performance liquid chromatography (HPLC), using the Ultimate 3000 liquid chromatograph (Thermo Scientific Dionex, Sunnyvale, CA, USA) equipped with a Refractive Index (IR) detector and the data processed in the Chromeleon 7.2 software (Thermo Scientific Dionex, Sunnyvale, CA, USA). The samples were injected onto a Bio‐Rad Aminex HPX‐87H column (300 × 7.8 mm, 9 µm; Hercules, CA, USA), operated at 50°C, using 5.0 mM H_2_SO_4_ as the mobile phase at a flow rate of 0.6 mL min^−1^ and a run time of 20 min. Sugar standards (glucose, arabinose, mannose, galactose, and xylose) were used to identify the saccharide components in the samples by comparing the retention times of the detected constituents with those of the standards.

### 2.5 Assessing the Potential Prebiotic of CWB

Using the method yielding the highest yield, the prebiotic activity of the extracted carbohydrates was determined from the freeze‐dried samples obtained in accordance with item 2.4.

#### 2.5.1 Evaluation of In Vitro Digestibility

To assess the in vitro digestibility of the CWB, the samples were subjected to simulated human digestion using an adaptation of the procedure described by Minekus et al. (2014). The prebiotics inulin and oligofructose were used as positive controls for comparative purposes. Solutions of simulated salivary fluid (SSF), simulated gastric fluid (SGF), and simulated intestinal fluid (SIF) were prepared according to Supporting Information Table S1. At different concentrations (15 and 30 g L^−1^), the samples were mixed with SSF in the presence of a salivary α‐amylase solution at 75 U mL^−1^; the pH of the medium was adjusted to 7.0 with 1 M NaOH and maintained with agitation at 37°C for 2 min at 130 rpm. SGF was added at a 1:1 ratio to a porcine pepsin solution (2000 U mL^−1^); the pH was adjusted to 3.0 with 1 M HCl and maintained with agitation at 130 rpm for 2 h at 37°C. SIF in a 1:1 ratio, a solution of pancreatin 800 U mL^−1^ and porcine bile extract (in the quantity required to reach 10 mM in the final mixture) were added to the solutions, followed by stirring at 45 rpm for 2 h at 37°C. The mixtures were centrifuged for 5 min at 2750 g, and the supernatants were collected.

Total and reducing sugars were then quantified using the Antrona (Osborne and Voogt 1985) and Somogyi‐Nelson (Maldonade et al. 2013) methods, respectively, and the degree of hydrolysis (H%) of the digested samples (Mohd Nor et al. 2017) was calculated according to Equation (2).

(2) $$H\%=\frac{\mathrm{reducing}\;\mathrm{sugar}\;\mathrm{released}}{\mathrm{total}\;\mathrm{sugar}\;\mathrm{content}-\mathrm{inicial}\;\mathrm{reducing}\;\mathrm{sugar}\;\mathrm{content}}.\;100$$

#### 2.5.2 Effect of CWB on the Cell Viability of Probiotic Microorganisms

The probiotic strains *B. lactis*, *L. acidophilus*, *L. paracasei*, and *L. rhamnosus* were used to evaluate the effect of CWB on the cell viability of probiotic microorganisms. For comparative purposes, the growth on glucose and prebiotics (inulin and oligofructose) in the appropriate media was also tested. Activation of the *Lactobacillus* spp. and *B. lactis* strains was carried out by inoculation in De Man, Rogosa, and Sharpe (MRS) broth (37°C, 20–24 h), followed by centrifugation (4500 g, 15 min, 4°C), washing and resuspension in sterile saline solution to obtain a suspension with counts of approximately 7 Log UFC mL^−1^ (Lacerda Massa et al. 2020; Rokana et al. 2017).

The viability of the microorganisms was determined by counting viable cells using MRS broth with a modified composition concerning the carbon source as a basal medium, as described by de Albuquerque et al. (2020), with modifications, where different broths were prepared: without a carbon source, containing 15 and 30 g L^−1^ of glucose (non‐prebiotic ingredient), containing 15 and 30 g L^−1^ of inulin (prebiotic), containing 15 and 30 g L^−1^ of oligofructose (prebiotic), containing 15 and 30 g L^−1^ of CWB.

Aliquots of the suspensions of the activated strains (2% v v^−1^) were inoculated into MRS broth (37°C, anaerobiosis), and at different time intervals (0, 24, and 48 h), aliquots of 1 mL of each mixture were serially diluted in sterile peptone solution and subsequently sown in depth on MRS agar (37°C, 72 h, anaerobiosis) to count the viable cells (Log CFU mL^−1^) the probiotic strains. Anaerobic conditions were obtained in an anaerobic jar (Permution, Curitiba, PR, Brazil) containing an anaerobic generator (Anaerobac, Probac, Santa Cecília, SC, Brazil).

#### 2.5.3 Determination of Prebiotic Activity Scores (PASs)

The PAS on each probiotic was determined by Equation (3). The PAS was determined in relation to enteric strains of *E. coli* and *S*. Typhimurium as described by Lacerda Massa et al. (2020).

(3) $$\mathrm{PAS}=\left(\frac{P24-P0}{G24-G0}\right)\;\rho -\left(\frac{P24-P0\;}{G24-G0}\right),e$$

where “*ρ*” indicates probiotic bacteria, “е” indicates enteric bacteria, *P* is the growth rate of the evaluated microorganism (Log CFU mL^−1^) when the prebiotic substrate is used as a sugar source at times 24 and 0 h, and *G* is the growth rate of the evaluated microorganism (Log CFU mL^−1^) when the non‐prebiotic substrate is used as a sugar source at times 24 and 0 h.

The enteric strains were activated by inoculation in brain heart infusion (BHI) broth (37°C, 18–20 h) followed by centrifugation (4500 × g, 15 min, 4°C), washing and resuspension in sterile saline solution to obtain a suspension with counts of approximately 7 Log CFU mL^−1^ (Lacerda Massa et al. 2020; Rokana et al. 2017).

Aliquots of the suspensions of the activated strains (2% v v^−1^) were inoculated into the nutrient broth (37°C, aerobiosis) with a modified composition concerning the carbon source as a basal medium, where different culture media were prepared: without a carbon source, containing 15 and 30 g L^−1^ of glucose (non‐prebiotic ingredient), containing 15 and 30 g L^−1^ of inulin (prebiotic), containing 15 and 30 g L^−1^ of oligofructose (prebiotic), containing 15 and 30 g L^−1^ of CWB; and at different time intervals (0, 24, and 48 h), 1 mL aliquots of each mixture were serially diluted in sterile peptone solution, and subsequently surface sown on MacConkey agar (44°C, 72 h, aerobiosis) to count viable cells (Log CFU mL^−1^) of the enteric strains (Supriatin et al. 2021).

The metabolic activity of the probiotic strains was assessed by determining the pH (Method 981.12; AOAC 2023) and the sugar and organic acid levels by HPLC (de Albuquerque et al. 2020), in the different culture media and at different incubation times (0, 24, and 48 h). The sugar standards used were xylose, mannose, arabinose, and glucose, and the acids were lactic and acetic (ASTM 2020).

### 2.6 Statistical Analysis

All tests were carried out randomly. The results of the experimental designs were analyzed using the *Protimiza Experimental Design* software, and the prebiotic activity evaluation tests were analyzed using *Spotfire Statistica* version 14 software. The adequacy of the models was assessed using analysis of variance (ANOVA), and Tukey's test was used to evaluate differences between means; *p*‐values ≤ 0.05 were considered statistically significant.

## 3 Results and Discussion

### 3.1 Centesimal Composition of WB

The proximate composition of WB reported in previous studies is presented in Table 1, together with the results obtained for the WB used as raw material in the present study.

**TABLE 1:** Proximate composition of wheat bran (WB) reported in previous studies and of the WB used in the present work.

Moisture	Ash	Protein	Lipids	Carbohydrates	Author
12.10	3.40–8.10	13.20–18.40	3.50–3.90	56.80	Apprich et al. (2014)
12	—	9.6–18.60	3–4	60–75	Prückler et al. (2014)
9.89	5.79	15.60	4.25	64.50	Li et al. (2023)
10.70	4.70	18.10	3.30	64.20	Xu et al. (2023)
11.28 ± 0.01	5.0 ± 0.02	13.73 ± 0.11	4.45 ± 0.07	65.53 ± 1.32	Paesani et al. (2024)
12.19 ± 0.10	4.03 ± 0.26	16.05 ± 0.08	5.53 ± 0.19	62.20 ± 0.26	This work

The moisture values found for the WB applied in this study were similar to those found by Apprich et al. (2014) and Prückler et al. (2014). The ash content found was in line with Apprich et al. (2014). The results for proteins and lipids varied more than those of the other authors. Carbohydrates showed results higher than those reported by Prückler et al. (2014) and Apprich et al. (2014) and lower than those reported by Li et al. (2023), Xu et al. (2023), and Paesani et al. (2024). The studies analyzed showed varying levels of protein (9.6%–18.6%) and carbohydrates (56.8%–75%), the latter being the component present in the greatest concentration in WB.

The variation in the values of the parameters analyzed (moisture, ash, proteins, lipids, and carbohydrates) described by each author can be explained by the influence of the different genetic varieties of wheat and/or environmental conditions of cultivation, processing, abrasion and the grinding process (Z. Chen et al. 2024; Khalid et al. 2023; Saini et al. 2023; Paesani et al. 2024).

### 3.2 Extraction of Carbohydrates From WB by Ultrasonic Treatment

In the first experimental design (CCRD 2^3^), the concentration of extracted carbohydrates ranged from 7.27 to 16.63 g CHO·100 g^−^^1^ WB. The quadratic model of carbohydrate concentration as a function of the variables is represented in Equation (3), and the variance analysis (ANOVA) had an *R* ^2^ 84.53%.

(3) $$\begin{array}{ccc} \left[\mathrm{CHO}\right](g\mathrm{of}\mathrm{CHO}.100g^{-1}\mathrm{of}\mathrm{WB}) & = & 11.58+1.26{x_{1}}^{2}+0.71{x_{2}}^{2}\\ & & +0.86x_{3}-1.50{x_{3}}^{2}. \end{array}$$

The solids load variable (linear term) had no significant effect on the response of extracted carbohydrates, despite the fact that Zheng et al. (2016) found that decreasing the solid load in suspension for extraction resulted in a greater difference in concentration between the external solvent (water) and the internal tissues of the bran, which would result in rapid dissolution of the polysaccharides and an increase in yield. Therefore, the variable study range was redefined from 30 to 100 g.L^−1^ to 20 to 50 g.L^−1^ for the next experimental design.

The time variable had a significant, positive effect, indicating an increase in the extracted carbohydrate content as this parameter increased from the lowest level (4 min) to the highest (9 min). This can be explained by the fact that ultrasound induces acoustic cavitation, and the exposure time contributes positively to the greater generation of microbubbles that collapse with each other, breaking their cellular structure and allowing the polysaccharides to diffuse into the medium (Chemat et al. 2017; Cui et al. 2021; W. Zhang et al. 2023; Z. ‐H. Chen et al. 2024). Therefore, the study range for this variable was redefined from 4 to 9 min to 11 to 14 min for the next experimental design.

The power amplitude variable did not have a significant effect, but Cui et al. (2021) found that ultrasound power is also related to extraction yield, where low‐power ultrasound devices result in lower yields than high‐power devices. Therefore, the power was set at 80% for the next stage of experimental design.

To maximize the extraction of carbohydrates from WB by ultrasonic treatment, considering the variables solids load (24 to 46 g.L^−1^) and extraction time (11 to 14 min), a CCRD 2^2^ was applied. The power amplitude was set at 80%.

The concentration of extracted carbohydrates was between 15.53 and 29.95 g CHO. 100 g^−1^ of WB. These values are higher than in the first experimental design, demonstrating that the variable's ranges were correctly adjusted.

Comparing the percentage of carbohydrates extracted (29.95%) with the values obtained in the centesimal composition analysis for the carbohydrate content of WB (62.2%), it can be said that there was a yield in the extraction process of 48.16%, which is lower than that found by Ferreira (2018) and Antunes et al. (2023) who also used ultrasonic treatment to extract carbohydrates from rice bran. However, it is higher than that reported by Z. ‐H. Chen et al. (2024), who used this methodology to extract polysaccharides from *Rosa roxburghii* bagasse for 60 and 180 min, yielding 3.87% and 4.01%, respectively. A direct comparison across different materials is limited, as the authors did not find any previous studies under similar conditions. The other studies that evaluated carbohydrate extraction from WB are discussed in Section 3.4.

### 3.3 Extraction of Carbohydrates From WB by Hydrothermal Treatment

First, a CCRD 2^3^ was carried out to evaluate the effects of thee independent variables. The concentration of extracted carbohydrates ranged from 10.54 to 44.49 g CHO. 100 g^−1^ of WB.

The variables did not have a significant effect (*p* ≥ 0.05) on the response of extracted carbohydrates; therefore, the time was set at 30 min to save energy, and the range of the solids load variable was reduced to 24 to 46 g.L^−1^ because, although it was insignificant, it had a negative effect. The pH variable had a negative effect, although it was insignificant; the study range was adjusted to 3.0–5.0 because Paesani et al. (2024) reported higher yields of soluble non‐starch polysaccharides from WB under more acidic conditions.

To improve results, the variable ranges were redefined to run a CCDR 2^2^. The concentration of extracted carbohydrates ranged from 23.68 to 48.02 g of CHO. 100 g^−1^ of WB. This result was higher than the previously obtained value (44.49%), but the *R* ^2^ from ANOVA was 64.86%, indicating that the model did not fit the data. However, the selected condition should be interpreted as the best experimental result within the tested range, rather than a predictive optimum.

Comparing the percentage of carbohydrates extracted (48.02%), with the values obtained in the centesimal composition analysis for the carbohydrate content of WB (62.2%), it can be said that there was a yield in the extraction process of 72.20%, a value very close to that found by Ferreira (2018) who also used hydrothermal treatment to extract carbohydrates from rice bran and obtained a yield of 78.30%. Other research evaluating the extraction of carbohydrates from WB is discussed in Section 3.4.

### 3.4 Yield of Extraction of Carbohydrates From WB by Ultrasonic and Hydrothermal Treatment

In the WB extracts obtained by ultrasound (WBU) and hydrothermal treatment (WBH), under the conditions that yielded the best carbohydrate concentration, a new extraction was performed for validation, and the process yield was calculated. The results are shown in Supporting Information Table S2. Extraction by hydrothermal treatment proved to be more efficient than ultrasound‐assisted extraction in terms of both carbohydrate quantification (45% and 27.04%, respectively) and extraction yield (72.36% and 43.47%, respectively), where the yields are higher than those found by other authors who used WB as the raw material for extraction.

The mechanical effects generated by acoustic cavitation during ultrasonication may enhance extraction by promoting cell disruption, particle size reduction, increased solvent penetration, and improved mass transfer (Kavindya et al. 2026; Wang et al. 2014). However, under the evaluated conditions, ultrasound treatment may have shown limited effectiveness in disrupting the compact lignocellulosic matrix of WB and promoting extensive carbohydrate release (Kavindya et al. 2026).

In hydrothermal processing, the combination of elevated temperature and pressure promotes water ionization, increasing the concentration of hydronium ions (H_3_O^+^), which favors the hydrolysis and depolymerization of hemicelluloses and other structural polysaccharides. This effect may be further enhanced by the release of acetyl groups and uronic acids during biomass deconstruction (Gallina et al. 2018; Gírio et al. 2018; Tripathi et al. 2022). However, a detailed structural characterization of the extracted carbohydrates, such as molecular weight distribution and degree of polymerization, was not performed in this study, limiting a more detailed mechanistic interpretation.

Shang et al. (2021) extracted WB polysaccharides using hydrothermal treatment at 90°C for 5 h, followed by alcohol precipitation and reported a lower extraction yield (16.09%) than that obtained in the present study, despite the longer extraction time. The research by Surin et al. (2020) shows that the variables temperature, time, solvent, and extracted material are significant parameters for extraction efficiency and method.

Given its higher carbohydrate content and extraction yield, together with the simpler operation and solvent‐free nature of the process, hydrothermal treatment was selected for subsequent characterization and in vitro prebiotic evaluations.

### 3.5 Characterization of CWB

The carbohydrates appeared as a white powder. Their appearance after precipitation and centrifugation, as well as after freeze‐drying and grinding, is shown in Supporting Information Figure S1.

#### 3.5.1 Functional Groups by FTIR Spectroscopy

**FIGURE 1:**
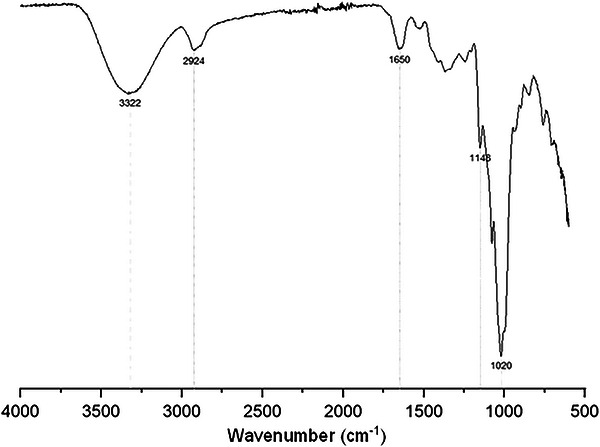
FTIR spectra of carbohydrates extracted from wheat bran by hydrothermal treatment.

The FTIR spectra of carbohydrates extracted from WB by hydrothermal treatment are shown in Figure 1. Characteristic absorptions can be seen in the region of 3322 cm^−1^ of the band attributed to the broad O‐H stretch, indicating the presence of hydroxyl groups, and along with the C = O carbonyl stretch at 1650 cm^−1^, indicating the presence of a carboxylic acid, as well as other oxygen‐containing groups (Nandiyanto et al. 2019; Wong et al. 2020). According to Silverstein (2019), polysaccharides usually have many OH groups, which have broad and intense bands near 3300 cm^−1^ due to the stretching of the O‐H bond.

At the same time, the peaks located at 1143 and 1020 cm^−1^ are characteristic of polysaccharide vibrations, attributed to the carbohydrate rings, as well as the CO, CC, and COC groups (Martins et al. 2022) and the peak at 2924 cm^−1^ is associated with the C‐H stretching mode (symmetric and asymmetric) generated from the ‐CH and ‐CH_2_ groups (Lafi et al. 2024).

These results show the selectivity of the extraction processes since carbohydrates were the only components present in the sample, with no peaks being identified that indicate other organic materials, such as lipids (with characteristic peaks between 1745 and 1725 cm^−1^) and proteins (two peaks between 1700 and 1500 cm^−1^; Nandiyanto et al. 2019; Bilal et al. 2024).

#### 3.5.2 Identification and Quantification of Carbohydrates by HPLC

Chromatographic analysis of the hydrolyzed extracts (Supporting Information Table S3 and Figure S2) showed that CWB is a mixture of carbohydrates rather than purified AXs, which may influence microbial utilization. The main sugar present was glucose, followed by arabinose. The predominance of glucose may reflect residual starch and other glucan‐derived components. However, this is according to Lv et al. (2021), who emphasize that glucose is the main monosaccharide in WB extracted with hot water for two times (3 h for each time) at 60°C and may also contain small amounts of arabinose and xylose. Nandini and Salimath (2001) analyzed the composition of water‐soluble carbohydrates in WB and found 79.2% glucose, 11.1% arabinose, and 8.4% xylose. This difference was attributed to the authors' different extraction and quantification methods, underscoring that, in this study, the focus was on higher yield, simplicity, and a solvent‐free approach.

### 3.6 Evaluation of the Prebiotic Activity of CWB

#### 3.6.1 Evaluation of In Vitro CWB Digestibility

The degree of hydrolysis of CWBs and standard prebiotics (inulin and oligofructose) determined after in vitro digestibility at different concentrations (15 and 30 g.L^−1^) is shown in Table 2. It is important to note that these concentrations were defined in accordance with ANVISA (2019), which lists the nutrients with standardized claims and their specific requirements and, for both inulin and fructooligosaccharides, indicates that the ingredient should provide at least 5 g and not exceed 30 g in the daily recommendation of the ready‐to‐eat product. Therefore, the maximum dosage representing an upper‐limit scenario (30 g) and the intermediate dosage (15 g) were considered. It corresponds approximately to 1.5–7.5 g, which is within the daily recommendation for a beverage or portion of 100–250 mL.

**TABLE 2:** Percentage of carbohydrate hydrolysis after in vitro digestibility.

Carbohydrates	Concentration (g L^−1^)	Hydrolysis degree (%)
Inulin	15 30	3.16 ± 0.38^c^ 2.39 ± 0.10^d^
Oligofructose	15 30	22.25 ± 0.91^a^ 18.53 ± 0.53^b^
CWB	15 30	0.86 ± 0.01^e^ 2.04 ± 0.11^d^

*Note*: Mean (±standard deviation, *n* = 3) followed by the same letters within a column are not significantly different (Tukey's test, *p* > 0.05).

Abbreviation: CWB, carbohydrates extracted from WB.

The degree of hydrolysis showed a direct relationship with concentration, with higher initial carbohydrate concentrations in the CWB (15–30 g L^−^^1^) promoting greater hydrolysis. However, this relationship was inverse for samples containing inulin and oligofructose.

Based on the results of the digestibility study, it can be deduced that when CWB is consumed at a concentration of 15 g L^−1^, around 99.14% of the product remains unchanged after exposure to the simulated gastrointestinal conditions in vitro, a higher percentage, compared to inulin (96.84%) and oligofructose (77.75%). However, when the initial concentration is increased to 30 g L^−1^, the percentage of unchanged product at the end of the simulated digestive process decreases to approximately 97.96%, 97.61%, and 81.47% for CWB, inulin, and oligofructose, respectively.

To evaluate the digestibility of inulin, Kumar et al. (2020) subjected inulin to contact with an artificial fluid in the intestinal (final) stage for 5 h; when they evaluated it at 2 h, the percentage of hydrolysis of inulin was approximately 6%, and at the end of 5 h, it reached 8.9%, values which are higher than those found in this study.

The result for inulin digestibility observed in this work was also lower than that found by Gunarathne et al. (2024), who found that the hydrolysis of inulin in contact with a simulated gastric fluid (for 6 h at pH 1) was 11%.

For inulin and oligofructose, the digestibility results were higher than those found by Antunes et al. (2023) or inulin (0.45% and 4.29%) and oligofructose (4.29% and 5.53%) at concentrations of 15 and 30 g L^−1^, respectively.

Higher results were also found by Ellegård et al. (1997) who added inulin to a controlled diet and analyzed the effluent collected by ileostomy, observing digestibility results of 12% for inulin. However, when they added oligofructose, the same authors reported a digestibility of 11%, which was lower than in the present study.

Nobre et al. (2018) tested commercial oligofructose at 12.5% and used the same methodology as in this study to assess digestibility, reporting a total digestibility of 4.9%.

The digestibility of oligofructose observed in this work was also higher than that found by de Figueiredo et al. (2020), who found that the hydrolysis of oligofructose in contact with enzymes present in the gastrointestinal tract after 4 h of incubation at 37°C was 21%.

The CWB digestibility results were higher than those reported by Amrein et al. (2003), who isolated one of the layers of the wheat grain, called aleurone, which comprises WB, and subjected it to in vitro digestibility analysis, finding overall digestibility of 32%. It is worth noting that the aleurone layer contains bioactive polysaccharides, including straight‐chain AXs, ferulic acids, antioxidants, and β‐glucans (Evers and Millar 2002).

It can therefore be observed that the percentage of hydrolysis is strongly influenced by the analytical methodology employed. Considering that one of the main requirements for classifying an ingredient as a dietary prebiotic is its resistance to hydrolysis by digestive enzymes and gastric acid, allowing it to reach the intestine and selectively stimulate beneficial microorganisms, the results indicate that most of the CWB fraction remained available for utilization by resident probiotic bacteria, particularly *Lactobacillus* and *Bifidobacterium* (Gibson et al. 2017; Mohd Nor et al. 2017).

#### 3.6.2 Effect of Carbohydrates on the Cell Viability of Probiotic Microorganisms

**FIGURE 2:**
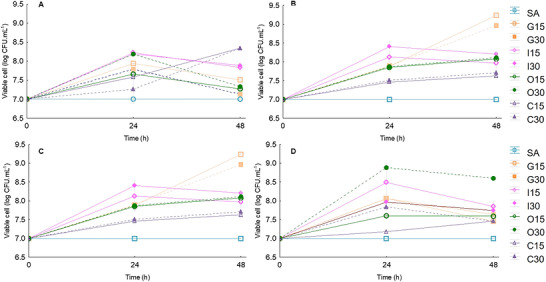
Viable cell counts of *L. acidophilus* (A), *L. paracasei* (B), *L. rhamnosus* (C), and *B. lactis* (D) in media with glucose (G15–15 g.L^−1^; G30–30 g.L^−1^), inulin (I15–15 g.L^−1^; I30–30 g.L^−1^), oligofructose (O15–15 g.L^−1^; O30–30 g.L^−1^), carbohydrates extracted from wheat bran (C15–15 g.L^−1^; C30–30 g.L^−1^) or without a sugar source (SA) during a 48 h of cultivation. Mean ± standard deviation, *n* = 3.

The viability of the microorganisms *L. acidophilus*, *L. paracasei, L. rhamnosus*, and *B. lactis* in basal broth containing or not containing the different sugar sources for 48 h is shown in Figure 2 (A—D).

The media without sugars did not support increased probiotic counts over time, demonstrating that each added sugar source is associated with the potential for bacterial growth (Antunes et al. 2023). Glucose was used as a readily fermentable reference substrate to support the interpretation of microbial responses.

The counts of the probiotics in the different media during the cultivation period were ≥ 7.0 Log CFU mL^−1^. All the probiotics tested showed counts of 7.1 to 9.3 Log CFU mL^−1^ in glucose media; 7.7 to 8.2 Log CFU mL^−1^ in inulin media; 7.3 to 8.6 Log CFU mL^−1^ in oligofructose media, and 7.3 to 8.3 Log CFU mL^−1^ in CWB media after 48 h of cultivation.

At 24 h of cultivation, for *L. paracasei, L. rhamnosus*, and *L. acidophilus*, CWB generated the lowest growth of the strains concerning glucose and the prebiotics tested; however, for *B. lactis*, CWB at a concentration of 30 g.L^−1^ provided greater growth than the medium containing 15 g.L^−1^ of oligofructose. For *L. rhamnosus, L. acidophilus*, and *L. paracasei*, the greatest growth was in media containing inulin; for *B. lactis*, the most significant growth was in media containing 30 g.L^−1^ of oligofructose.

At 48 h of cultivation, for *L. paracasei, L. rhamnosus*, and *B. lactis*, CWB generated the lowest growth of the strains concerning glucose and the prebiotics tested. For *L. rhamnosus* and *L. paracasei*, the highest growth was observed in media containing glucose, which can be attributed to glucose being a monosaccharide and therefore more fermentable than complex carbohydrates (Antunes et al. 2023; Teberga 2018).

For *L. acidophilus*, the best performance was in media containing CWB at concentrations of 15 and 30 g.L^−1^, reaching ≈ 8.3 Log CFU mL^−1^ after 48 h of cultivation (rate of ≈ 1.3 Log CFU mL^−1^), similar to that found by Marotti et al. (2012) who, when evaluating the growth of *L. plantarum* L12 in different varieties of durum wheat, obtained counts ranging from ≈ 7.9 to 8.2 Log CFU mL^−1^ after 48 h.

According to Huebner et al. (2007), an essential requirement for a carbohydrate to be considered prebiotic is that it is metabolized by a probiotic in a similar or superior way to glucose. Therefore, by observing the count of the probiotic *L. acidophilus* during cultivation in CWB media, we can indicate that it has the ability to metabolize WB carbohydrates as a growth substrate, thus suggesting its potential prebiotic effect (de Albuquerque et al. 2020; Antunes et al. 2023; Huebner et al. 2007; Lacerda Massa et al. 2020).

#### 3.6.3 PASs

The PAS values for *E. coli* ATCC 43888 and *S*. Typhimurium ATCC 14028 are shown in Table 3.

**TABLE 3:** Prebiotic activity scores of inulin, oligofructose, CWB for *L. acidophilus, L. paracasei, L. rhamnosus*, and *B. lactis* on *S*. Typhimurium.

Enteric strains	Sugar source	Concentration	Microrganisms			
			*L. acidophilus*	*L. paracasei*	*L. rhamnosus*	*B. lactis*
*Salmonella* Typhimurium	Inulin	15 g L^−1^	0.77 ± 0.02^Ab^	0.38 ± 0.01^Ac^	−0.05 ± 0.00^Ad^	1.16 ± 0.00^Aa^
	Oligofructose		0.14 ± 0.06^Cb^	0.40 ± 0.02^Aa^	−0.18 ± 0.01^Ac^	0.00 ± 0.00^Cbc^
	CWB		−0.46 ± 0.01^Eb^	−0.56 ± 0.00^Cb^	−0.03 ± 0.04^Aa^	−0.91 ± 0.01^Ec^
	Inulin	30 g L^−1^	0.12 ± 0.03^CDa^	0.19 ± 0.10^Ba^	−0.53 ± 0.02^Bb^	−0.47 ± 0.17^Db^
	Oligofructose		−0.06 ± 0.04^Da^	−0.57 ± 0.02^Cb^	−0.75 ± 0.09^Bb^	0.22 ± 0.03^BCa^
	CWB		0.34 ± 0.01^Bbc^	0.27 ± 0.01^Bc^	0.00 ± 0.00^Ad^	0.48 ± 0.06^Ba^
*Escherichia coli*	Inulin	15 g L^−1^	−0.79 ± 0.02^Ac^	−1.18 ± 0.01^Ab^	−1.61 ± 0.00^Ba^	−0.41 ± 0.00^Ad^
	Oligofructose		−1.43 ± 0.06^Bb^	−1.17 ± 0.02^Ac^	−1.75 ± 0.01^Ba^	−1.57 ± 0.00^Bab^
	CWB		−1.51 ± 0.01^Bb^	−1.61 ± 0.00^Bb^	−1.09 ± 0.04^Ac^	−1.96 ± 0.01^Ba^
	Inulin	30 g L^−1^	−4.65 ± 0.03^Cb^	−1.18 ± 0.02^Cb^	−5.31 ± 0.02^Ca^	−5.24 ± 0.17^Da^
	Oligofructose		−4.66 ± 0.04^Cb^	−5.17 ± 0.02^Da^	−5.35 ± 0.09^Ca^	−4.38 ± 0.03^Cb^
	CWB		−5.50 ± 0.01^Dbc^	−5.58 ± 0.01^Eb^	5.85 ± 0.00^Da^	−5.37 ± 0.06^Dc^

*Note*: Mean ± standard deviation (*n* = 3); mean followed by the same small letters within a row are not significantly different (Tukey's test, *p *> 0.05); mean followed by the same capital letters within a column are not significantly different (Tukey's test, *p* > 0.05).

Abbreviation: CWB, carbohydrates extracted from WB.

A positive PAS indicates that the carbohydrate selectively promotes probiotic growth relative to the non‐prebiotic reference substrate (glucose), while a negative PAS suggests reduced selectivity and lower stimulation of probiotic growth in comparison with the non‐prebiotic reference (de Albuquerque et al. 2020; Kumar et al. 2020; Lacerda Massa et al. 2020).

In respect of *E. coli*, the PAS of the CWB was negative and lower (*p* ≤ 0.05) than the standard prebiotics (inulin and oligofructose), regardless of concentration for *L. acidophilus, L. paracasei*, and *B. lactis*. However, for *L. rhamnosus*, despite being negative, the PAS was higher (*p* ≤ 0.05) than for inulin and oligofructose at a concentration of 15 g.L^−1^. These results are in disagreement with Paesani et al. (2024), who evaluated the prebiotic activity of soluble non‐starch polysaccharides from WB extracted by different chemical treatments and obtained positive PAS in all treatments; and for Marotti et al. (2012), who evaluated the prebiotic activity in other varieties of durum wheat and also found positive values, except for the *Senatore Cappelli* wheat variety in which the score was −0.01, but, despite being negative, it was higher than that found in this research.

This study's PAS for inulin and oligofructose was lower than that reported in previous research. These differences may be due to the source of inulin and oligofructose and the methodology used (Huebner et al. 2007; Kumar et al. 2020; NithyaBalaSundari et al. 2020; Rubel et al. 2014; Shalini et al. 2017). The results obtained for the CWB indicate that the carbohydrate has no prebiotic activity against *E. coli*.

The PAS values for *S*. Typhimurium were lower and negative for CWB at 15 g L^−^^1^ for *L. acidophilus*, *L. paracasei*, and *B*. *lactis* (*p* ≤ 0.05) when compared with the standard prebiotics. For *L. rhamnosus*, no statistical difference was observed (*p* ≥ 0.05). However, at 30 g L^−^^1^, PAS values obtained with CWB were higher than those of oligofructose for L. paracasei (*p* ≤ 0.05) and similar to those of inulin (*p* ≥ 0.05). For *B*. *lactis*, PAS values with CWB were comparable to oligofructose (*p* ≥ 0.05) and higher than inulin (*p* ≤ 0.05). In the cases of *L. rhamnosus* and *L. acidophilus*, CWB showed positive PAS values that were higher than those observed for inulin and oligofructose (*p* ≤ 0.05). In particular, CWB at 30 g L^−^^1^ showed positive PAS values for *L. acidophilus* relative to *S*. Typhimurium, indicating a more favorable selective response under these conditions. Together with the higher cell counts and metabolic activity observed for *L. acidophilus* (Section 3.6.2), these findings support the prebiotic potential properties of CWB (Lacerda Massa et al. 2020), although similar responses were not consistently observed for the other probiotic enteric combinations.

The PAS is intrinsically dependent on the metabolic characteristics and substrate utilization pathways of each microorganism evaluated (Figueroa‐González et al. 2019). Studies have demonstrated substantial interspecies variability in CAZyme profiles and oligosaccharide utilization mechanisms among intestinal microorganisms, enteric and probiotics, even within closely related taxa, which may explain differences observed in PASs (Goh and Klaenhammer 2015; Wardman et al. 2022; N. Zhang et al. 2022).

Therefore, a compound considered potentially prebiotic may promote the growth and metabolic activity of one microorganism, while showing limited or no effect on another. This was verified in this study, as CWB showed better results when PAS was evaluated in relation to *S*. Typhimurium rather than *E. coli*. This selective stimulation is consistent with the current concept of prebiotics, which recognizes microorganism‐specific responses rather than a universal effect across all intestinal bacteria (Rastall and Gibson 2015).

Another hypothesis to explain these results is the possible co‐extraction of other carbohydrates, mainly starch, which may have influenced microbial utilization patterns. Although starch was not quantified in the present study, its presence cannot be ruled out because no specific pretreatment step was applied to remove it. Previous studies investigating WB‐derived prebiotic fractions commonly included a starch‐removal step before extraction (Aguedo et al. 2014; Maes and Delcour 2002; Nandini and Salimath 2001; Paesani et al. 2024; Sun et al. 2019). Therefore, the possible presence of residual starch should be considered a limitation of this work. Nevertheless, the objective of the present study was to evaluate a simplified, potentially scalable extraction strategy without extensive pretreatment steps.

In fact, further structural characterization—including molecular weight distribution and degree of polymerization—is necessary to establish clear structure–function relationships and to enable more direct comparisons with well‐defined prebiotics such as inulin and oligofructose.

#### 3.6.4 Parameters of the Metabolic Activity of Probiotics

##### 3.6.4.1 Sugar Content

The concentrations of arabinose, glucose, mannose, and xylose during cultivation of the probiotic strains in media containing glucose, inulin, oligofructose, or CWB are shown in Table 4. Xylose and mannose were also quantified because the AXs of WB, which have been suggested to have a prebiotic action (Cloetens et al. 2010; Gill et al. 2021), are hemicelluloses that have a xylose structure with arabinose side‐lines (Marotti et al. 2012); and Shang et al. (2021) analyzed the monosaccharide composition of WB and found arabinose, xylose, mannose, and glucose.

**TABLE 4:** Concentration of the arabinose (ARA), glucose (GLU), and mannose plus xylose (M+X) in media with glucose (G15: 15 g.L^−1^; G30: 30 g.L^−1^), inulin (I15: 15 g.L^−1^; I30: 30 g.L^−1^), oligofructose (O15: 15 g.L^−1^; O30: 30 g.L^−1^), carbohydrates extracted from WB (C15 – 15 g.L^−1^; C30 – 30 g.L^−1^) or without a sugar source (NS), inoculated with *L. acidophilus*, *L. paracasei*, *L. rhamnosus*, and *B. lactis*.

Sugar	Sugar source	*L. acidophilus*			*L. paracasei*			*L. rhamnosus*			*B. lactis*		
		0 h	24 h	48 h	0 h	24 h	48 h	0 h	24 h	48 h	0 h	24 h	48 h
ARA	NS	0.53 ± 0.04^G^	—	—	0.55 ± 0.01^G^	—	—	—	—	—	3.71 ± 0.01^F^	—	—
	G15	12.60 ± 0.01^Ba^	11.95 ± 0.00^Ab^	—	12.60 ± 0.01^Ba^	11.95 ± 0.00^Ab^	—	0.51 ± 0.01^D^	—	—	—	—	8.29 ± 0.01^Aa^
	G30	30.69 ± 0.01^Aa^	3.99 ± 0.02^Bb^	1.46 ± 0.01^Ac^	30.69 ± 0.01^Aa^	3.99 ± 0.02^Bb^	1.46 ± 0.01^Ac^	0.53 ± 0.01^C^	—	—	3.79 ± 0.01^Ea^	1.30 ± 0.01^Db^	0.75 ± 0.01^Dc^
	I15	3.99 ± 0.01^Ca^	0.60 ± 0.02^Eb^	0.50 ± 0.01^Cc^	3.99 ± 0.01^Ca^	0.59 ± 0.01^Eb^	.51 ± 0.01^Cc^	2.68 ± 0.01^A^	1.41 ± 0.01^Bb^	0.55 ± 0.01^c^	23.81 ± 0.01^Ba^	4.29 ± 0.01^Bb^	3.32 ± 0.02^Bc^
	I30	0.73 ± 0.02^F^	—	—	0.73 ± 0.02^F^	—	—	2.55 ± 0.01^Ba^	2.51 ± 0.01^A^	—	40.32 ± 0.01^Aa^	3.29 ± 0.01^Cb^	—
	O15	2.69 ± 0.01^Da^	1.24 ± 0.01^Db^	0.79 ± 0.01^Bb^	2.69 ± 0.01^Da^	1.24 ± 0.01^Db^	0.81 ± 0.01^Bc^	—	—	—	9.44 ± 0.01^Da^	4.31 ± 0.01^Bb^	—
	O30	1.99 ± 0.01^Ea^	1.41 ± 0.02^Cb^	—	2.00 ± 0.01^Ea^	1.41 ± 0.01^Cb^	—	—	—	—	16.54 ± 0.02^Ca^	5.81 ± 0.01^Ab^	1.01 ± 0.01^Cc^
	C15	—	—	—	—	—	—	—	—	—	2.43 ± 0.01^H^	—	—
	C30	0.78 ± 0.03^F^	—	—	0.78 ± 0.03^F^	—	—	—	—	—	3.19 ± 0.01^G^	—	—
GLU	NS	—	—	—	—	—	—	2.79 ± 0.02^Ba^	2.23 ± 0.21^ABb^	—	1.12 ± 0.12^E^	—	—
	G15	—	—	—	—	—	—	2.53 ± 0.11^Ca^	2.46 ± 0.06^Aa^	2.30 ± 0.14^Aa^	1.51 ± 0.13^DE^	—	—
	G30	0.50 ± 0.00^B^	—	—	0.50 ± 0.00^B^	—	—	2.77 ± 0.08^Ba^	2.38 ± 0.07^ABab^	2.04 ± 0.15^Ab^	1.75 ± 0.10^Dea^	1.20 ± 0.12^Bb^	—
	I15	—	—	—	—	—	—	6.90 ± 0.14^Aa^	1.60 ± 0.07^CDEb^	1.14 ± 0.20^Bb^	11.13 ± 0.18^B^	—	—
	I30	—	—	—	—	—	—	2.71 ± 0.07^Ba^	1.87 ± 0.14^BCDb^	1.17 ± 0.13^Bc^	25.33 ± 0.47^Aa^	21.3 ± 0.42^Ab^	—
	O15	—	—	—	—	—	—	2.91 ± 0.13^Ba^	1.16 ± 0.10^EFb^	0.86 ± 0.14^Bb^	2.26 ± 0.23^D^	—	—
	O30	—	—	—	—	—	—	2.12 ± 0.12^Ca^	1.00 ± 0.15^Fb^	0.74 ± 0.14^Bc^	6.16 ± 0.22^C^	—	—
	C15	1.33 ± 0.14^A^	—	—	1.52 ± 0.12^A^	—	—	2.71 ± 0.13^Ba^	2.11 ± 0.16^ABCa^	1.14 ± 0.20^Bb^	—	—	—
	C30	—	—	—	—	—	—	2.89 ± 0.16^Ba^	1.49 ± 0.15^DEFb^	1.10 ± 0.14^Bb^	—	—	—
M + X	NS	0.73 ± 0.09^B^	—	—	0.67 ± 0.16^A^	—	—	—	—	—	—	—	—
	G15	0.88 ± 0.16^Ba^	0.63 ± 0.10^Aa^	—	0.81 ± 0.16^Aa^	0.52 ± 0.16^Aa^	—	6.40 ± 0.^14Ba^	1.74 ± 0.08^Bb^	—	—	—	—
	G30	0.66 ± 0.11^B^	—	—	0.51 ± 0.17^A^	—	—	15.6 ± 0.^15Aa^	9.09 ± 0.13^Ab^	5.12 ± 0.12^c^	0.73 ± 0.13^F^	—	—
	I15	0.85 ± 0.11^B^	—	—	0.70 ± 0.13^Aa^	0.50 ± 0.14^Aa^	—	1.49 ± 0.15^Ca^	0.41 ± 0.12^Cb^	—	15.17 ± 0.24^Ba^	8.10 ± 0.14^Bb^	—
	I30	2.06 ± 0.14^Aa^	0.72 ± 0.09^Ab^	—	0.49 ± 0.21^A^	—	—	1.61 ± 0.15^C^	—	—	21.53 ± 0.15^Aa^	16.0 ± 0.23^Ab^	—
	O15	0.70 ± 0.14^B^	—	—	0.47 ± 0.21^A^	—	—	—	—	—	1.69 ± 0.10^Ea^	0.39 ± 0.15^DEb^	—
	O30	0.44 ± 0.15^B^	—	—	—	—	—	0.50 ± 0.15^D^	—	—	4.43 ± 0.09^Da^	2.42 ± 0.09^Cb^	—
	C15	0.42 ± 0.14^B^	—	—	0.46 ± 0.19^A^	—	—	—	—	—	4.55 ± 0.16^Da^	0.40 ± 0.14^DEb^	—
	C30	0.70 ± 0.11^B^	—	—	0.53 ± 0.18^A^	—	—	—	—	—	6.79 ± 0.13^Ca^	0.47 ± 0.04^Db^	—

*Note*: Mean (± standard deviation, *n* = 3) followed by the same capital letters for the same probiotic strain, at the same time are not significantly different (Tukey's test, *p* > 0.05). Mean (± standard deviation, *n* = 3) followed by the same small letters for the same probiotic strain, in the same culture media, and at different times are not significantly different (Tukey's test, *p* > 0.05).

All sugars were partially or completely consumed during incubation, regardless of the culture medium or the probiotic strain used.

Arabinose was not detected in broth containing oligofructose or CWB, regardless of concentration or cultivation time (0, 24, or 48 h) when inoculated with *L. rhamnosus*, except for broth containing glucose, which, at time 0, had a concentration of ≈ 0.5 g.L^−1^ of this sugar. When inoculated with *B. lactis*, the inulin‐containing media had a higher initial arabinose concentration and were consumed over time. After 48 h, arabinose was quantified in the media containing glucose (in both concentrations), inulin (at a concentration of 15 g.L^−1^), and oligofructose (at a concentration of 30 g.L^−1^), with the sugar remaining between 0.7 and 8.3 g.L^−1^. The media inoculated with *L. acidophilus* and *L. paracasei* had similar behavior; in both cases, the highest concentrations of arabinose were in the media containing glucose at time 0 h, and at the end of the cultivation time, this sugar was reduced from ≈ 30.6 to ≈ 1.5 g.L^−1^ in the media containing 30 g.L^−1^ of glucose.

Glucose was detected in all media inoculated with *L. rhamnosus*, regardless of the sugar source. Glucose concentrations decreased over time, regardless of culture media, but remained similar (*p* ≥ 0.05) between 24 and 48 h, except for inulin and oligofructose at 15 g.L^−1^, which differed significantly at 48 h, compared to 24 h. In the media inoculated with *B. lactis*, glucose was not detected in the media containing CWB but was detected in the other media, with greater evidence in the media containing inulin. After 24 h, glucose was detected only in the media containing 30 g.L^−1^ of glucose and inulin, and it was completely consumed within 48 h. Media inoculated with *L. acidophilus* and *L. paracasei* showed similar results, with glucose detected only at 0 h in media containing 30 g.L^−1^ glucose and 15 g.L^−1^ CWB.

Mannose and xylose were combined because the chromatographic conditions used did not allow separation of these peaks for quantification. The concentrations of these sugars decreased over time, regardless of the culture media and strain inoculated. They were detected in all the media inoculated with *L. acidophilus* at 0 h, most clearly in the media containing 30 g.L^−^^1^ of inulin. They did not differ in the other media (*p* ≥ 0.05), being completely consumed over time. In media inoculated with *L. paracasei*, the sugars did not differ at time zero (*p* ≥ 0.05) and were consumed entirely over time; however, in the media containing 30 g.L^−1^ of oligofructose, these sugars were not detected. In the media inoculated with *L. rhamnosus*, mannose and xylose were detected most clearly in the media containing 30 g.L^−1^ of glucose and were only partially consumed over the cultivation time. For the media containing 15 g.L^−1^ of glucose and inulin and 30 g.L^−1^ of inulin and oligofructose, the sugars were consumed entirely after 48 h of cultivation. Media inoculated with *B. lactis* did not show concentrations of mannose and xylose in the media without sugar and with 15 g.L^−1^ of glucose. The highest concentration of these sugars was observed in the media containing inulin; as in the other media, they were completely consumed over time.

The decrease in arabinose, glucose, mannose, and xylose content in CWB media indicates its use to promote an increase in the population of *L. acidophilus* and *L. paracasei*, over time (Lacerda Massa et al. 2020), which contributes to the idea that WB carbohydrates can be used as growth substrates for these probiotic strains.

##### 3.6.4.2 pH Evolution and Acid Production

**FIGURE 3:**
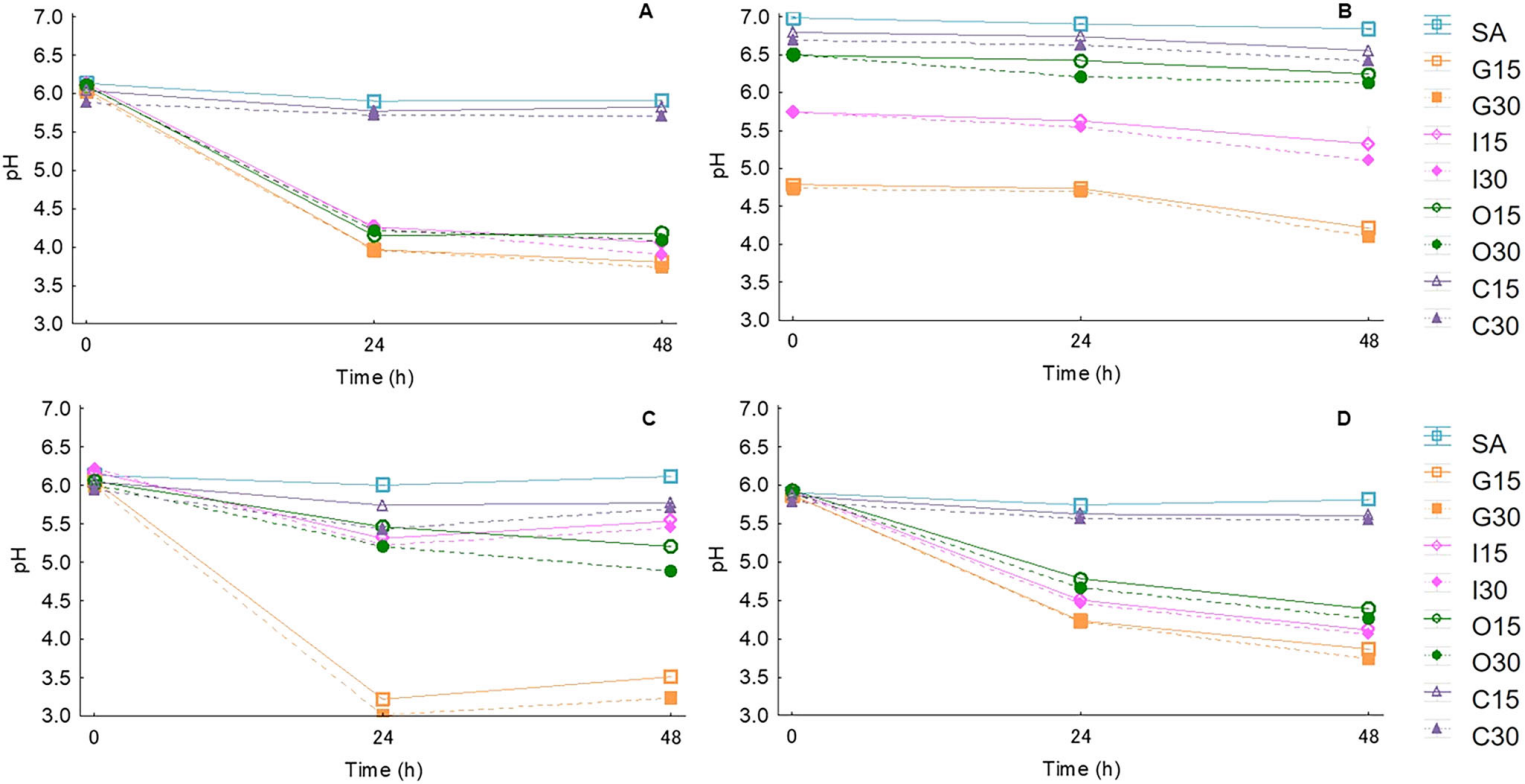
pH evaluation in media with glucose (G15–15 g.L^−1^; G30–30 g.L^−1^), inulin (I15–15 g.L^−1^; I30–30 g.L^−1^), oligofructose (O15–15 g.L^−1^; O30–30 g.L^−1^), carbohydrates extracted from wheat bran (C15–15 g.L^−1^; C30–30 g.L^−1^) or without a sugar source (SA), inoculated with *L. acidophilus* (A), *L. paracasei* (B), *L. rhamnosus* (C), and *B. lactis* (D) during 48 h. Mean ± standard deviation, *n* = 3.

Cultivating probiotics in media with different sugar sources resulted in decreases in pH over time (Figure 3A–D). The acidifying activity of the probiotic strains increased with increasing sugar concentration. The highest pH was observed between 24 and 48 h in media without sugar, and the lowest was in media containing glucose, regardless of the probiotic strain. All the probiotic strains promoted a lower pH reduction (*p* ≤ 0.05) in media with added CWB (∆pH ≤ 0.28) when compared to media with the other sugars (∆pH ≥ 0.66) at the end of the cultivation time.

The contents of acetic and lactic acids during cultivation (Table 5) increased over time, regardless of the cultivation media. In general, lactic acid was the most produced organic acid (*p* ≤ 0.05) in all the probiotic strains tested after 48 h of incubation.

**TABLE 5:** Concentration of acid acetic and lactic in media with glucose (G15 – 15 g.L^−1^; G30 – 30 g.L^−1^), inulin (I15 – 15 g.L^−1^; I30 – 30 g.L^−1^), oligofructose (O15 – 15 g.L^−1^; O30 – 30 g.L^−1^), carbohydrates extracted from WB (C15 – 15 g.L^−1^; C30 – 30 g.L^−1^) or without a sugar source (NS), inoculated with *L. acidophilus*, *L. paracasei*, *L. rhamnosus*, and *B. lactis*.

Acidis	Sugar source	*L. acidophilus*			*L. paracasei*			*L. rhamnosus*			*B. lactis*		
		0 h	24 h	48 h	0 h	24 h	48 h	0 h	24 h	48 h	0 h	24 h	48 h
Acetic	NS	—	—	4.21 ± 0.01^Ba^	—	—	0.74 ± 0.01^Ea^	—	—	—	—	—	5.45 ± 0.01^Fa^
	G15	—	—	3.61 ± 0.01^Ca^	—	—	—	—	8.42 ± 0.01^Bb^	11.21 ± 0.01^Ba^	—	5.12 ± 0.00^Bb^	8.79 ± 0.01^Ca^
	G30	3.14 ± 0.01^Bc^	4.10 ± 0.01^Ab^	25.41 ± 0.01^Aa^	—	—	1.65 ± 0.01^Ca^	0.97 ± 0.02^Ac^	10.66 ± 0.01^Ab^	12.52 ± 0.01^Aa^	2.11 ± 0.01^c^	4.11 ± 0.01^Db^	6.34 ± 0.02^Da^
	I15	3.81 ± 0.01^Ac^	—	—	—	1.62 ± 0.01^Ab^	1.81 ± 0.00^Ba^	0.64 ± 0.01^Bc^	0.65 ± 0.01^Eb^	0.76 ± 0.02^Ea^	—	—	—
	I30	3.16 ± 0.01^Bc^	3.76 ± 0.01^Bb^	3.87 ± 0.02^Ca^	0.91 ± 0.01^c^	1.07 ± 0.01^Bb^	1.19 ± 0.01^Da^	—	0.61 ± 0.02^Fb^	0.86 ± 0.01^Da^	—	2.01 ± 0.01^Gb^	4.09 ± 0.01^Ha^
	O15	—	—	—	—	—	—	—	1.34 ± 0.01^Ca^	1.36 ± 0.01^Ca^	—	4.79 ± 0.01^Cb^	17.99 ± 0.01^Ba^
	O30	—	2.83 ± 0.00^Cb^	—	—	—	3.69 ± 0.01^Aa^	—	0.80 ± 0.01^Da^	0.84 ± 0.01^Da^	—	5.75 ± 0.01^Ab^	18.83 ± 0.01^Aa^
	C15	—	—	—	—	—	—	—	—	—	—	2.19 ± 0.01^Fb^	6.11 ± 0.01^Ea^
	C30	—	—	—	—	—	—	—	—	—	—	4.04 ± 0.02^Eb^	5.32 ± 0.02^Ga^
Lactic	NS	1.24 ± 0.02^Fc^	1.30 ± 0.01^Hb^	3.98 ± 0.00^Ia^	1.24 ± 0.02^Fc^	2.54 ± 0.02^Eb^	3.14 ± 0.01^Ea^	26.55 ± 0.01^Ac^	27.52 ± 0.03^Cb^	32.06 ± 0.01^Aa^	—	—	31.7 ± 0.02^Aa^
	G15	2.50 ± 0.01^Dc^	4.16 ± 0.01^Eb^	4.79 ± 0.00^Ha^	0.86 ± 0.01^Ic^	1.45 ± 0.01^Ib^	1.70 ± 0.01^Ia^	23.43 ± 0.01^Fc^	23.92 ± 0.02^Fb^	25.71 ± 0.01^Ha^	5.42 ± 0.01^Ac^	16.33 ± 0.01^Ab^	21.03 ± 0.00^Ca^
	G30	3.05 ± 0.02^Cc^	4.39 ± 0.01^Db^	6.51 ± 0.01^Ga^	0.99 ± 0.02^Hc^	1.57 ± 0.01^Hb^	1.77 ± 0.02^Ha^	16.90 ± 0.01^Hc^	22.58 ± 0.03^Gb^	27.94 ± 0.02^Ga^	^−^	—	—
	I15	18.01 ± 0.02^Ac^	23.69 ± 0.02^Ab^	24.26 ± 0.01^Ba^	18.04 ± 0.02^Bc^	22.47 ± 0.02^Ab^	23.68 ± 0.02^Aa^	22.43 ± 0.04^Gc^	26.91 ± 0.02^Db^	30.33 ± 0.02^Ea^	4.16 ± 0.00^Cc^	11.43 ± 0.01^Bb^	20.86 ± 0.01^Da^
	I30	3.64 ± 0.01^Bc^	19.41 ± 0.02^Bb^	21.80 ± 0.00^Da^	19.65 ± 0.02^Ac^	20.85 ± 0.02^Bb^	21.08 ± 0.04^Da^	24.26 ± 0.01^Dc^	25.92 ± 0.02^Eb^	30.00 ± 0.01^Fa^	4.06 ± 0.01^Dc^	9.14 ± 0.01^Cb^	16.84 ± 0.01^Fa^
	O15	—	1.98 ± 0.01^Gb^	15.51 ± 0.01^Fa^	1.73 ± 0.01^Dc^	9.21 ± 0.01^Db^	21.34 ± 0.01^Ca^	24.48 ± 0.02^Cc^	26.99 ± 0.02^Db^	—	4.31 ± 0.01^Bc^	8.54 ± 0.01^Db^	19.89 ± 0.03^Ea^
	O30	0.83 ± 0.00^Gc^	2.31 ± 0.02^Fb^	16.23 ± 0.01^Ea^	2.02 ± 0.02^Cc^	9.43 ± 0.02^Cb^	22.25 ± 0.00^Ba^	25.82 ± 0.03^Bc^	28.04 ± 0.06^Bb^	30.85 ± 0.01^Ca^	3.38 ± 0.01^Ec^	6.99 ± 0.02^Eb^	25.49 ± 0.01^Ba^
	C15	0.70 ± 0.01^Hc^	4.69 ± 0.01^Cb^	23.49 ± 0.02^Ca^	1.35 ± 0.01^Ec^	1.74 ± 0.02^Gb^	2.20 ± 0.02^Ga^	23.77 ± 0.01^Ec^	26.03 ± 0.04^Eb^	30.79 ± 0.02^Da^	—	1.99 ± 0.02^Gb^	3.01 ± 0.01^Ga^
	C30	1.93 ± 0.02^Ec^	4.73 ± 0.01^Cb^	26.51 ± 0.02^Aa^	1.09 ± 0.01^Gc^	1.88 ± 0.02^Fb^	2.31 ± 0.01^Fa^	25.75 ± 0.00^Bc^	30.24 ± 0.01^Ab^	31.32 ± 0.02^Ba^	2.02 ± 0.03^Fc^	2.08 ± 0.02^Fa^	2.61 ± 0.02^Ha^

*Note*: Mean (± standard deviation, *n* = 3) followed by the same capital letters for the same probiotic strain, at the same time are not significantly different (Tukey's test, *p* > 0.05). Mean (± standard deviation, *n* = 3) followed by the same small letters at the same for the same probiotic strain, in the same culture media and at different times are not significantly different (Tukey's test, *p* > 0.05).

Lactic acid, although not grouped together as short‐chain fatty acids (SCFA), is one of the most important end products of glucose and fructose fermentation by *Lactobacillus* and *Bifidobacterium* and can be produced in the colon during carbohydrate fermentation (Markowiak‐Kopeć and Śliżewska 2020; Lacerda Massa et al. 2020), which justifies the increase (*p* ≤ 0.05) in the concentration of this acid in all media over time, regardless of the probiotic inoculated.

Acetic acid was produced in higher concentration in the broth containing glucose inoculated with *L. acidophilus*, and in the broths containing oligofructose incubated with *B. lactis*, which is under expectations because, according to the literature, it is an important metabolite formed by *Lactobacillus* and *Bifidobacterium* during colonic fermentation of carbohydrates (de Albuquerque et al. 2020; Lacerda Massa et al. 2020).

The increased production of lactic and acetic acids over time, together with sugar consumption (Section 3.6.4.1), higher probiotic counts in CWB media, and the associated decrease in pH, is a desirable finding in the investigation of candidate prebiotic ingredients, as it indicates intense metabolic activity and has been associated with beneficial effects, including inhibition of enteric pathogens and anti‐inflammatory and immunoregulatory actions (Aleman and Yadav 2023; de Oliveira et al. 2024; Xiong et al. 2022). In particular, the metabolic behavior observed for *L. acidophilus* in CWB media suggests active utilization of the extracted carbohydrates. The progressive reduction in sugar concentration, together with increased cell counts, indicates substrate consumption associated with microbial growth. This response was accompanied by increased production of lactic and acetic acids and a consequent decrease in pH, supporting the occurrence of intense fermentative metabolism. These combined responses reinforce the ability of CWB to sustain probiotic activity and support its prebiotic potential properties.

## 4 Conclusion

Hydrothermal extraction provided the highest carbohydrate yield (72.36%) from WB. The extracted carbohydrates showed resistance to simulated gastrointestinal digestion and supported the growth and metabolic activity of probiotic strains, with the strongest evidence observed for *Lactobacillus acidophilus*. Positive PASs were mainly observed for L. acidophilus in relation to *Salmonella* Typhimurium, whereas responses for the remaining probiotic strains and enteric comparisons were more limited. These findings indicate that WB‐derived carbohydrates exhibit prebiotic potential properties and may represent candidate prebiotic ingredients, although further in vivo studies and broader validation are required to confirm their functionality.

## Author Contributions

**Débora Regina Magro**: investigation, methodology, formal analysis, data curation, writing – original draft. **Maria Eduarda Cordeiro Silva**: formal analysis, investigation. **Deisy Alessandra Drunkler**: conceptualization, investigation, funding acquisition, writing – review and editing. **Flavio Dias Ferreira**: investigation, methodology, formal analysis. **Eliane Colla**: conceptualization, investigation, funding acquisition, writing – original draft, methodology, validation, visualization, writing – review and editing, formal analysis, project administration, supervision, resources.

## Conflicts of Interest

The authors declare no Conflicts of Interest.

## Supporting information




**Supporting Information Tables**: jfds71280‐sup‐0001‐Tables.docx

**Supporting Information Figures**: jfds71280‐sup‐0002‐Figures.docx
