# Supplementary material for: Wheat Bran‐Derived Carbohydrates as Functional Food Ingredients: Extraction and Evaluation of Prebiotic Potential
Source: J Food Sci. 2026 Jul 9;91(7):e71280. doi: 10.1111/1750-3841.71280 (PMC13347277; doi:10.1111/1750-3841.71280)
Supplement: Supplementary file 1 — Supporting Information Tables: jfds71280‐sup‐0001‐Tables.docx [file JFDS-91-0-s002.docx]

Suplementary Table 1: Preparation of stock solutions of Simulated Salivary Fluid (SSF), Simulated Gastric Fluid (SGF) and Simulated Intestinal Fluid (SIF)

| Constituent | FSS | | FGS | | FIS | |
| --- | --- | --- | --- | --- | --- | --- |
|  | **Concentration (mmol/L)** | **Volume**  **(mL)** | **Concentration (mmol/L)** | **Volume**  **mL** | **Concentration (mmol/L)** | **Volume**  **mL** |
| KCl | 15.1 | 15.1 | 6.9 | 6.9 | 6.8 | 6.8 |
| KH_2_PO_4_ | 3.7 | 3.7 | 0.9 | 0.9 | 0.8 | 0.8 |
| NaHCO_3​_ | 6.8 | 13.6 | 12.5 | 25 | 42.5 | 85 |
| NaCl | - | - | 11.8 | 47.2 | 9.6 | 38.4 |
| MgCl_2_(H_2_O)_6_​ | 0.5 | 0.15 | 0.4 | 0.1 | 1.1 | 0.33 |
| (NH_4_)2CO_3_​​ | 0.06 | 0.06 | 0.5 | 0.5 | - | - |

Author: Minekus et al*.,* 2014

The volumes are calculated for a final volume of 500 mL for each simulated fluid.

Supplementary Table 2: carbohydrate concentration and yield of wheat bran extraction by ultrasound (WBU) and hydrothermal treatment (WBH)

| Test | Carbohydrate concentration (g of CHO.100 g^-1^ of WB) | Extraction yield (%) |
| --- | --- | --- |
| WBU | 27.04 ± 1.29^b^ | 43.47 ± 2.08^b^ |
| WBH | 45.00 ± 1.71^a^ | 72.36 ± 2.75^a^ |

Mean (±standard deviation, n = 3) followed by the same letters within a column are not significantly different (Tukey's test, p > 0.05).

Supplementary Table 3: Sugar composition of the hydrolysate by HPLC

| Sugars | Retention time (min) | Concentration | |
| --- | --- | --- | --- |
|  |  | **(g.L^-1^)** | **% total** |
| Glucose | 8.3 | 32.42 ± 0.03^a^ | 89.81 ± 0.08 |
| Arabinose | 11.6 | 3.68 ± 0.02^b^ | 10.19 ± 0.06 |

Mean ± standard deviation (n = 3 followed by the same letters within a column are not significantly different (Tukey’s test, p > 0.05).
