# Supplementary material for: Wheat Bran‐Derived Carbohydrates as Functional Food Ingredients: Extraction and Evaluation of Prebiotic Potential
Source: J Food Sci. 2026 Jul 9;91(7):e71280. doi: 10.1111/1750-3841.71280 (PMC13347277; doi:10.1111/1750-3841.71280)

Supplementary Figure 1 – carbohydrates extracted from wheat bran by hydrothermal treatment after precipitation and centrifugation (a) and after freeze-drying and grinding (b).


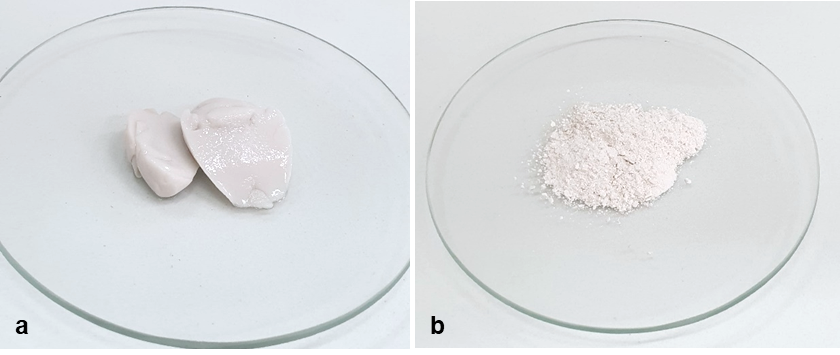


Supplementary Figure 2 – Chromatogram of hydrolysates obtained from carbohydrates extracted from wheat bran by hydrothermal treatment.


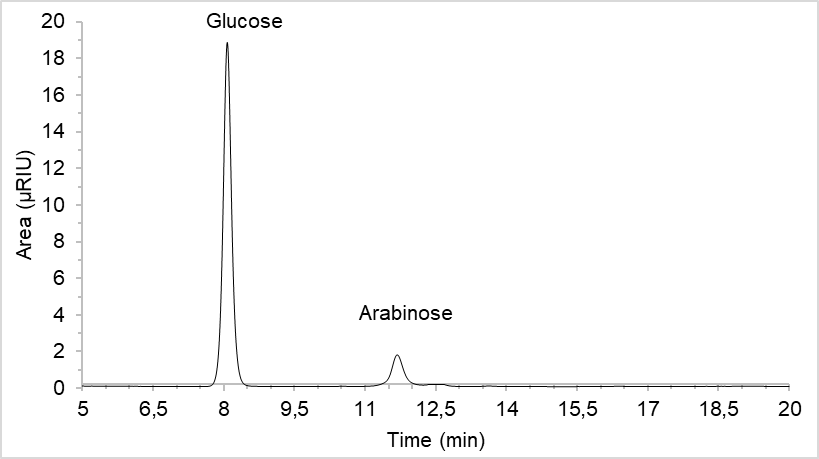

Supplement: Supplementary file 2 — Supporting Information Figures: jfds71280‐sup‐0002‐Figures.docx [file JFDS-91-0-s001.docx]
